# Cryogenic Bottom-up Formation of the Benzene Cation from Acetylene in Helium Nanodroplets

**DOI:** 10.1021/jacs.6c04278

**Published:** 2026-05-02

**Authors:** Florian Foitzik, Vincent Richardson, Colombe Maurice, Gabriel Schöpfer, Milan Ončák, Elisabeth Gruber

**Affiliations:** † Department of Ion Physics and Applied Physics, 27255 University of Innsbruck, Innsbruck 6020, Austria; ‡ Department of Physics, 4591 University of Liverpool, Liverpool L69 7ZX, United Kingdom; ¶ ENS Paris-Saclay, 329018 Université Paris-Saclay, Gif-sur-Yvette 91190, France

## Abstract

Bottom-up ion–molecule reaction networks are thought to drive molecular complexity in space. Nevertheless, even the formation of the first aromatic ring (benzene) remains poorly understood. In this publication, we report the formation of the benzene radical cation from an acetylene cation and two acetylene molecules via the sequential two-step reaction C_2_H_2_^•+^ + C_2_H_2_ → C_4_H_4_^•+^ followed by C_4_H_4_^•+^ + C_2_H_2_ → C_6_H_6_^•+^ inside cryogenic helium nanodroplets. The superfluid helium-environment, used as a model system to study chemical reactivity in an environment with efficient energy dissipation such as interstellar dust and ice, stabilizes reaction intermediates and products and enables direct spectroscopic investigation. Helium-tagging spectroscopy in the visible wavelength range unambiguously identifies the C_6_H_6_^•+^ product as the benzene radical cation. This is consistent with quantum-chemical calculations that predict a reaction pathway that is barrierless in both steps. These results establish a low-temperature route to the formation of the first aromatic ring in very cold condensed phases, which may impact our understanding of polycyclic aromatic hydrocarbon formation in the interstellar medium.

## Graphical abstract

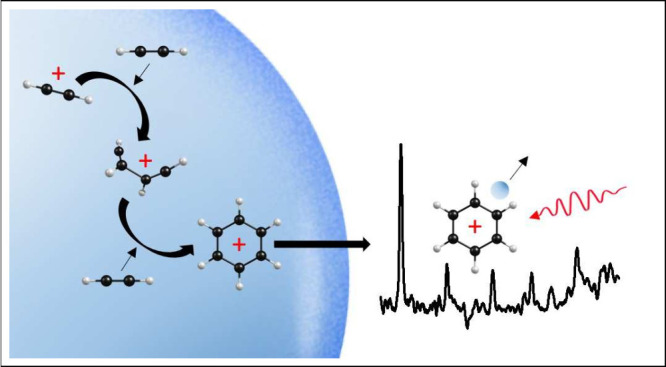

Although ions are less abundant than neutral species in the interstellar medium (ISM), ion–molecule reactions are central to its chemical evolution as they proceed efficiently even under low-temperature and low-density conditions.^1^ This is because ion–neutral reactions typically exhibit small or vanishing activation barriers, leading to significantly larger reaction rates than equivalent neutral–neutral processes. Ionization events driven by energetic processes such as cosmic rays and UV irradiation are therefore expected to initiate reaction networks,^2^ where reactions of atomic and small molecular ions can lead to the buildup of increasingly complex molecules.^3,4^ These ion-initiated processes are thereby anticipated to contribute significantly to the diverse range of molecules and ions that have so far been observed in interstellar environments.^5–7^

Among these species, the role of polycyclic aromatic hydrocarbons (PAHs) in the ISM and other astrochemical environments has been a subject of growing research interest. Not only are PAHs key components of interstellar dust,^8^ they have also been proposed to play an important role in prebiotic chemistry,^9^ and are thought to be the source of the aromatic infrared bands.^10,11^ While the simplest aromatic ring system, benzene (C_6_H_6_) has been detected via IR spectroscopy in the protoplanetary nebula CRL 618,^12^ as well as in protoplanetary disks by JWST,^13^ detection of PAHs has proven highly challenging. This can be attributed to the high molecular symmetry of many PAHs, which leads to the absence of a permanent electric dipole moment and consequently prevents their detection by rotational spectroscopy. However, a major breakthrough came with the detection of benzonitrile in 2018.^14^ The cyano functional group (−)­C≡N) introduces a permanent electric dipole moment, enabling the radio frequency detection of otherwise invisible aromatic systems. Since then, this approach has been applied to PAHs, leading to the identification of several cyano-substituted PAHs.^15–21^ In addition, other PAHs possessing small intrinsic permanent electric dipole moments have been detected via radio astronomy in cold dark clouds.^22–24^

Despite the importance of PAHs, their formation pathways remain incompletely characterized. The field continues to be highly active, with numerous neutral–neutral and ion–neutral reaction mechanisms for PAH growth proposed and investigated in recent years.^25–31^ However, the mechanisms leading to the formation of the first aromatic ring, benzene, remain unclear. A number of gas-phase mechanisms have been proposed, including ion–molecule,^32,33^ radical-molecule,^34,35^ neutral–neutral,^36^ and even transition metal-catalyzed reactions.^37^ While no definitive pathway has been experimentally confirmed in cold and isolated environments, among the many proposed mechanisms, acetylene (C_2_H_2_) and its derivatives are expected to play a significant role due to its widespread nature and combination of high reactivity and stability toward decomposition.^38^ Recently, however, a reactivity study performed using Coulomb crystals suggested that isolated bottom-up reactions of acetylene molecules terminate in an unreactive C_6_H_5_^+^ ion and therefore do not contribute to the formation of benzene,^39^ though these findings are currently the subject of ongoing debate.^40^ While these findings point to potential limitations of the isolated ion–acetylene pathway, the overall feasibility of astrochemical benzene formation under such conditions remains uncertain.

However, while ion reactivity studies have focused mainly on gas-phase processes, the role of ions in interstellar ices and at the gas–surface interface has received considerably less attention. Previous experimental studies on the irradiation of acetylene ices below its ionization threshold have reported the formation of benzene,^41–43^ yet the potential role of ions in these systems was not considered. Calculations have long predicted that reactions initiated by ion deposition on icy grain mantles could be an efficient source of complex molecules,^44,45^ with pioneering experimental measurements on the irradiation of amorphous water ice by low energy CH_3_^+^ ions demonstrating this to be an efficient method for methanol formation.^46^ While experimental measurements involving swift ions, and in particular swift heavy ions, are comparatively commonplace,^47,48^ experimental measurements of low-energy ions are still largely absent, despite their importance having been highlighted by calculations.^49^

Helium nanodroplets (HNDs) offer a promising platform to address this problem. These superfluid clusters of helium provide an ultracold (0.37 K) chemically inert matrix, in which ions and neutrals can be cotrapped, enabling the observation of low-temperature chemistry.^50^ The droplets can dissipate the excess energy generated by an exothermic reaction via evaporation of helium atoms from the droplet, stabilizing reaction intermediates or products that would otherwise fragment. Due to these properties, HNDs are often discussed in the context of nanoreactors^51^ and have been used to study chemical reactions of neutrals, as well as ions with neutrals in a solvent environment. HNDs have also been used as a perturbation-free environment to probe fundamental dynamics under controlled conditions.^52^ In particular, the efficient energy-dissipating inside HNDs makes them a model system that can mimic interstellar dust and ice environments in many ways.^53^ However, it should be noted that measurements within HNDs are only representative of the isolated interaction of a given reaction system, and cannot account for the interaction with the wider environments. Accurate modeling of the role of such interactions in astrochemical ices will therefore require experimental data on the diverse range of different reaction systems present, as well as consideration of the impact of different environments on the rate of energy dissipation, potentially impacting product fragmentation.

The ionization of acetylene clusters embedded in HNDs has recently been reported by Moon et al. to produce small covalently bound hydrocarbon cations C_4_H_*n*_^+^ (*n* = 2 – 5).^54^ Although ions corresponding to larger species, such as C_6_H_6_^•+^, appear at higher levels of acetylene doping, no spectroscopic evidence for aromatic ring formation has been identified. These results suggest that, although the initial steps of acetylene oligomerization can occur under ultracold, energy-dissipating conditions, the full reaction pathway and the structure of larger reaction products, such as C_6_H_6_^•+^, remain unclear and requires further investigation.

In this communication, we report findings on the sequential reaction of two neutral acetylene molecules with C_2_H_2_^•+^ inside HNDs. We report the formation of the benzene radical cation (C_6_H_6_^•+^), evidenced by its spectral signature in the visible wavelength range. In contrast to earlier studies, where the formation of cationic benzene via acetylene cyclotrimerization occurred as an intracluster reaction following ionization of neutral acetylene clusters,^55,56^ we demonstrate the formation of the benzene cation through the sequential addition of neutral acetylene molecules to an acetylene cation at low temperatures.

The experimental setup used in this work has been described in detail previously.^57^ Briefly, acetylene is doped into preionized multiply charged HNDs. Each charge center, distributed across the droplet surface,^58^ acts as a nucleation site for cluster growth, with the first acetylene molecule that arrives at a charge center being ionized by charge transfer from He^•+^. Additional neutral acetylene molecules are then sequentially pulled toward the charge centers by ion-induced dipole interactions, where they eventually undergo ion–molecule reaction. Collisional evaporation of the droplets through the addition of room-temperature helium buffer gas enables the extraction of the resultant ions, which are then detected using a time-of-flight mass-spectrometer.

Furthermore, by adjusting the helium pressure in the evaporation cell, the complexes can be prepared in a microsolvated helium environment, which ensures that the complexes remain cold upon extraction from the droplets. Under such conditions acetylene evaporation is prevented, which ensures that no fourth acetylene molecule was attached at any time to the trimer. Therefore, we conclude that the reaction products C_6_H_6_^•+^ are formed in bottom-up processes. The remaining helium-microsolvation also allows us to perform messenger spectroscopy of the ionic products. Upon resonant photoabsorption, the weakly bound helium atoms detach from the ions, and the resulting bare molecular ions are detected in a time-of-flight mass-spectrometer as a function of laser wavelength. A schematic of the overall experimental process is shown in Figure 1.

**Figure 1:**
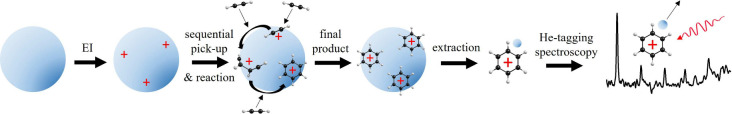
Schematic of the experimental process. Sequential ion–molecule reactions occur inside helium nanodroplets. The reaction products are extracted and characterized by He-tagging spectroscopy.

The vibronic spectrum of the *m*/*z* = 78 reaction product, corresponding to the mass of C_6_H_6_^•+^, was recorded from 18 200 cm^–1^ to 20 000 cm^–1^, which encompasses the vibronic bands of the X → B transition of the benzene cation.^59,60^ Figure 2 compares the spectrum of the reaction product of the trimerization of acetylene observed in our experiment (Figure 2a) with two reference spectra^59,60^ of the benzene cation from the literature (Figure 2b). The agreement of all observed absorption bands with the reference data unambiguously demonstrates that the reaction proceeds to yield the benzene cation. It should be noted, that the peak intensities in the work of Walter et al.^60^ might be misleading due to potential baseline problems as well as broader peak shapes compared to Goode et al.^59^ and the data presented in this work. In addition, quantum chemical calculations at the CAM-B3LYP/aug-cc-pVTZ level were performed using Gaussian 16 ^61^ to model the Franck–Condon spectrum of the benzene radical cation for the D_0_ → D_2_ transition (panel c of Figure 2). We note that quantum chemical modeling of the spectrum is nontrivial due to Jahn–Teller splitting, shallow potential energy surface, and possible multireference issues (see Supporting Information for details). Therefore, it is not surprising that the calculated spectrum matches the experimental one only qualitatively.

**Figure 2:**
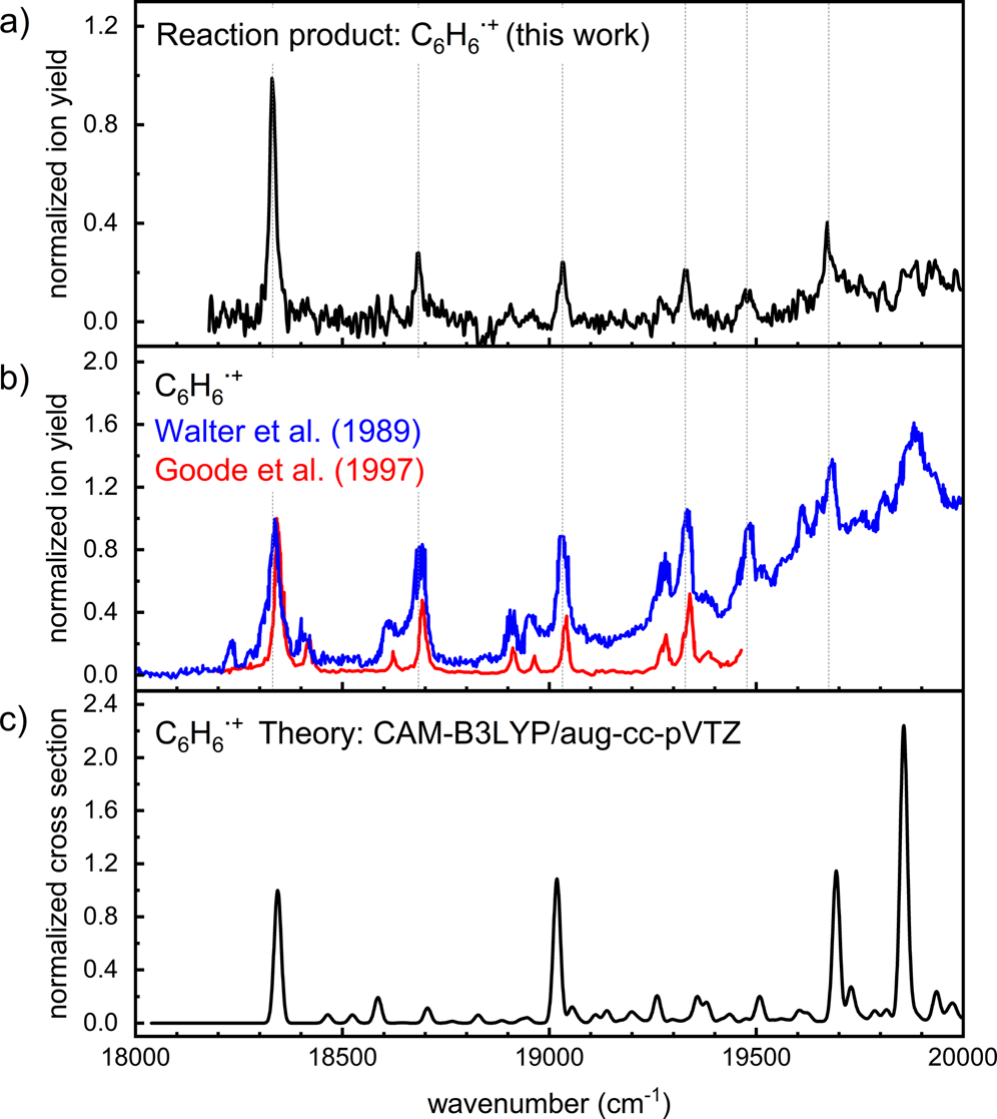
(a) Helium-tagging spectrum of the reaction product of the cationic trimerization of acetylene. (b) Literature spectra of the benzene radical cation.^59,60^ (c) Modeled Franck–Condon spectrum of the benzene radical cation at 0 K using quantum chemical calculations at the CAM-B3LYP/aug-cc-pVTZ level of theory. The calculated spectrum was shifted by −260 cm^–1^.

Since the sequential reaction takes place within ultracold helium nanodroplets, both addition steps must proceed with either very low, or without barriers entirely. To investigate this further, we have performed calculations of the reaction pathway at the CCSD­(T)/aug-cc-pVTZ//ωB97XD/aug-cc-pVTZ level of theory, as shown in Figure 3. The calculations predict the barrierless formation of a C_4_H_4_^•+^ intermediate, followed by the barrierless formation of an open C_6_H_6_^•+^ structure (minimum 1). Subsequent cyclization proceeds via two small barriers of 0.09 and 0.14 eV, yielding the benzene radical cation as the final product. From the spectra presented above we conclude that energy dissipation to the surrounding helium is not sufficiently fast to prevent progression over these extremely small barriers, even under such ultracold conditions.

**Figure 3:**
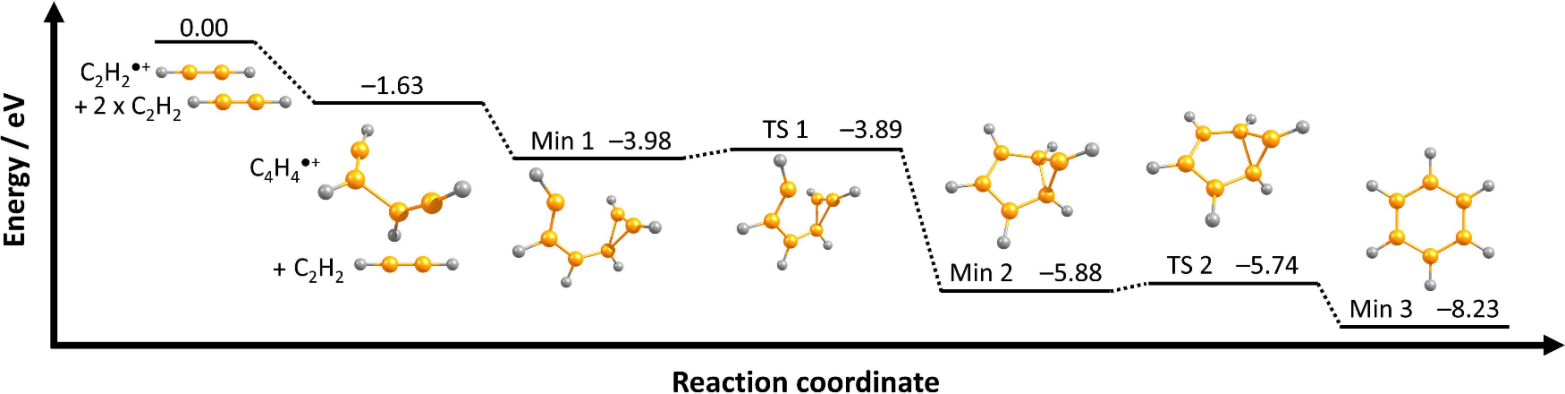
Reaction pathway for the formation of C_6_H_6_^•+^ by the sequential addition reaction of two units of C_2_H_2_ to C_2_H_2_^•+^ with relative energies in eV as calculated at the CCSD­(T)/aug-cc-pVTZ//ωB97XD/aug-cc-pVTZ level of theory (not to scale).

Importantly, however, while the total reaction C_2_H_2_^•+^ + 2C_2_H_2_ → C_6_H_6_^•+^ is exothermic by 8.23 eV, the dissipation of energy to the surrounding helium matrix is sufficiently efficient to prevent fragmentation. This represents a clear difference to previous observation in the gas-phase, where the reactions of both C_2_H_2_^•+^ and C_2_H_3_^+^ yield C_4_H_3_^+^ as the ionic product^39,62,63^ due to the inability to dissipate energy to the environment. This then leads to potentially unreactive C_6_H_5_^+^ as the product of the second sequential reaction,^39,64^ rather than the C_6_H_6_^•+^ product ion observed in this work.

While the rate of energy dissipation can be expected to vary between the cryogenic matrix used here and different solid surfaces, we note that only a small fraction of the energy released by the reaction needs to be dissipated for fragmentation to be energetically inaccesible. As a result, even comparatively inefficient energy dissipation to a surface can be expected to facilite the mechanism detailed above.

From this observation, we conclude that the sequential, matrix-mediated reaction of C_2_H_2_^•+^ with two acetylene molecules is a viable bottom-up formation pathway of cationic benzene, the first aromatic building block, under cold interstellar conditions. This marks a key step for the formation of larger aromatic species. The helium matrix used in this work is inert, which means that no catalytic activity is needed for the reaction to take place, potentially making a wide range of interstellar dust and ice grains suitable as similarly passive reaction matrices. However, the presence of other molecules can alter the reaction pathways and branching ratios. For example, in previous studies on mixed H_2_O/C_2_H_2_ ices,^65^ a series of products resulting from the formation of C–O bonds was observed, with the interactions between C_2_H_2_^•+^ and C_2_H_2_ accounting for only a small fraction of the overall chemistry of the low-energy ions. Therefore, additional experimental studies covering different chemical environments are needed to fully capture the diversity of astrochemical ices.

However, benzene radical cations formed through the reaction of C_2_H_2_^•+^ with C_2_H_2_ could undergo onward reaction or neutralization on the surface, or be evaporated to undergo further reactions in the gas-phase. This mechanism therefore represents a plausible first-step toward the formation of increasingly complex PAHs in the ISM.

## Supplementary Material



## Notes

Additional computational details and spectra computed with alternative methods and energy, structure, and vibrational frequencies of optimized structures (PDF)

## References

[1] Larsson M., Geppert W. D., Nyman G. (2012). Ion chemistry in space. Rep. Prog. Phys..

[2] Herbst E., Klemperer W. (1973). The formation and depletion of molecules in dense interstellar
clouds. Astrophys. J..

[3] Heays A. N., Bosman A. D., Van Dishoeck E. F. (2017). Photodissociation
and photoionisation
of atoms and molecules of astrophysical interest. Astron. Astrophys..

[4] Herbst E., Garrod R. T. (2022). Synthetic approaches
to complex organic molecules in
the cold interstellar medium. Front. Astron.
Space Sci..

[5] McGuire B. A. (2022). 2021 Census
of Interstellar, Circumstellar, Extragalactic, Protoplanetary Disk,
and Exoplanetary Molecules. Astrophys. J. Suppl.
S..

[6] Müller H. S., Thorwirth S., Roth D., Winnewisser G. (2001). The Cologne
database for molecular spectroscopy, CDMS. Astron.
Astrophys..

[7] Müller, H. S. P. ; Endres, C. P. ; Stutzki, J. ; Schlemmer, S. The Cologne Database for Molecular Spectroscopy. [Online], 2026; https://cdms.astro.uni-koeln.de.

[8] Gavilan
Marin L., Bejaoui S., Haggmark M., Svadlenak N., de Vries M., Sciamma-O’Brien E., Salama F. (2020). Low-temperature
formation of carbonaceous dust grains from PAHs. Astrophys. J..

[9] Ehrenfreund P., Rasmussen S., Cleaves J., Chen L. (2006). Experimentally tracing
the key steps in the origin of life: The aromatic world. Astrobiology.

[10] Schlemmer S., Cook D. J., Harrison J. A., Wurfel B., Chapman W., Saykally R. J. (1994). The unidentified interstellar infrared bands: PAHs
as carriers?. Science.

[11] Peeters E., Mackie C., Candian A., Tielens A. G. G. M. (2021). A Spectroscopic
View on Cosmic PAH Emission. Acc. Chem. Res..

[12] Cernicharo J., Heras A. M., Tielens A. G. G. M., Pardo J. R., Herpin F., Guélin M., Waters L. B. F. M. (2001). Infrared Space Observatory’s
Discovery of C_4_H_2_, C_6_H_2_, and Benzene in CRL 618. Astrophys. J..

[13] van
Dishoeck E. F., Grant S., Tabone B., Van Gelder M., Francis L., Tychoniec L., Bettoni G., Arabhavi A., Gasman D., Nazari P. (2023). The diverse chemistry
of protoplanetary disks as revealed by JWST. Faraday Discuss..

[14] McGuire B. A., Burkhardt A. M., Kalenskii S., Shingledecker C. N., Remijan A. J., Herbst E., McCarthy M. C. (2018). Detection of the
aromatic molecule benzonitrile (c-C_6_H_5_CN) in
the interstellar medium. Science.

[15] McGuire B. A., Loomis R. A., Burkhardt A. M., Lee K. L. K., Shingledecker C. N., Charnley S. B., Cooke I. R., Cordiner M. A., Herbst E., Kalenskii S. (2021). Detection of two interstellar polycyclic aromatic
hydrocarbons via spectral matched filtering. Science.

[16] Sita M. L., Changala P. B., Xue C., Burkhardt A. M., Shingledecker C. N., Lee K. L. K., Loomis R. A., Momjian E., Siebert M. A., Gupta D. (2022). Discovery of interstellar
2-cyanoindene (2-C_9_H_7_CN) in GOTHAM observations
of TMC-1. Astrophys. J. Lett..

[17] Cernicharo J., Cabezas C., Fuentetaja R., Agúndez M., Tercero B., Janeiro J., Juanes M., Kaiser R. I., Endo Y., Steber A. L. (2024). Discovery
of two cyano
derivatives of acenaphthylene (C_12_H_8_) in TMC-1
with the QUIJOTE line survey. Astron. Astrophys..

[18] Cernicharo J., Tercero B., Marcelino N., Lopez-Perez J. A., Gallego J. D., Tercero F., Esplugues G., Cabezas C., Agúndez M., Limeres C., Steber A. L., Perez D., Perez C., Lesarri A., de Vicente P. (2026). Discovery
of two new isomers of cyanoacenaphthylene (C_12_H_7_CN) in the Taurus molecular cloud 1 with the QUIJOTE line survey. Astron. Astrophys..

[19] Wenzel G., Cooke I. R., Changala P. B., Bergin E. A., Zhang S., Burkhardt A. M., Byrne A. N., Charnley S. B., Cordiner M. A., Duffy M. (2024). Detection of interstellar 1-cyanopyrene: A four-ring
polycyclic aromatic hydrocarbon. Science.

[20] Wenzel G., Speak T. H., Changala P. B., Willis R. H., Burkhardt A. M., Zhang S., Bergin E. A., Byrne A. N., Charnley S. B., Fried Z. T. P. (2025). Detections of interstellar aromatic nitriles
2-cyanopyrene and 4-cyanopyrene in TMC-1. Nat.
Astron..

[21] Wenzel G. (2025). Discovery of the Seven-ring Polycyclic Aromatic Hydrocarbon Cyanocoronene
(C_24_H_11_CN) in GOTHAM Observations of TMC-1. Astrophys. J. Lett..

[22] Cernicharo J., Agúndez M., Cabezas C., Tercero B., Marcelino N., Pardo J. R., de Vicente P. (2021). Pure hydrocarbon cycles in TMC-1:
Discovery of ethynyl cyclopropenylidene, cyclopentadiene and indene. Astron. Astrophys..

[23] Cabezas C., Agúndez M., Pérez C., Villar-Castro D., Molpeceres G., Pérez D., Steber A. L., Fuentetaja R., Tercero B., Marcelino N., Lesarri A., de Vicente P., Cernicharo J. (2025). Discovery of interstellar phenalene (c-C_13_H_10_): A new piece in the chemical puzzle of PAHs in space. Astron. Astrophys..

[24] Fuentetaja R., Cabezas C., Agúndez M., Tercero B., Marcelino N., de Vicente P., Cernicharo J. (2026). Discovery of 1H-cyclopent­[cd]­indene
(c-C_11_H_8_) in TMC-1 with the QUIJOTE line survey:
A new three-ringed polycyclic aromatic hydrocarbon. Astron. Astrophys..

[25] Soliman A. R., Attah I. K., Hamid A. M., El-Shall M. S. (2015). Growth kinetics
and formation mechanisms of complex organics by sequential reactions
of acetylene with ionized aromatics. Int. J.
Mass Spectrom..

[26] Zhao L., Kaiser R. I., Xu B., Ablikim U., Ahmed M., Evseev M. M., Bashkirov E. K., Azyazov V. N., Mebel A. M. (2018). Low-temperature
formation of polycyclic aromatic hydrocarbons in Titan’s atmosphere. Nat. Astron..

[27] Doddipatla S., Galimova G. R., Wei H., Thomas A. M., He C., Yang Z., Morozov A. N., Shingeldecker C. N., Mebel A. M., Kaiser R. I. (2021). Low-temperature gas-phase formation
of indene in the interstellar medium. Sci. Adv..

[28] Rap D. B., Schrauwen J. G. M., Marimuthu A. N., Redlich B., Brünken S. (2022). Low-temperature
nitrogen-bearing polycyclic aromatic hydrocarbon formation routes
validated by infrared spectroscopy. Nat. Astron..

[29] Rap D. B., Schrauwen J. G., Redlich B., Brünken S. (2024). Noncovalent
Interactions Steer the Formation of Polycyclic Aromatic Hydrocarbons. J. Am. Chem. Soc..

[30] Sutton P., Saunier J., Lao K. U., El-Shall M. S. (2024). Sequential Reactions
of Acetylene with the Benzonitrile Radical Cation: New Insights into
Structures and Rate Coefficients of the Covalent Ion Products. Chem. Lett..

[31] Arns R., McClish R., Hemberger P., Bodi A., Bouwman J., Kasper T., Schleier D. (2025). Is Phenylnitrene
a Missing Link in
the Formation of Polycyclic Aromatic Nitrogen Hetrocycles. Angew. Chem., Int. Ed..

[32] Stein T., Bandyopadhyay B., Troy T. P., Fang Y., Kostko O., Ahmed M., Head-Gordon M. (2017). Ab initio dynamics and photoionization
mass spectrometry reveal ion–molecule pathways from ionized
acetylene clusters to benzene cation. Proc.
Natl. Acad. Sci. U.S.A..

[33] McEwan M. J., Scott G. B. I., Adams N. G., Babcock L. M., Terzieva R., Herbst E. (1999). New H and H_2_ Reactions with Small Hydrocarbon
Ions and Their Roles in Benzene Synthesis in Dense Interstellar Clouds. Astrophys. J..

[34] Jones B. M., Zhang F., Kaiser R. I., Jamal A., Mebel A. M., Cordiner M. A., Charnley S. B. (2011). Formation of benzene in the interstellar
medium. Proc. Natl. Acad. Sci. U.S.A..

[35] Agúndez M., Cabezas C., Tercero B., Marcelino N., Gallego J. D., de Vicente P., Cernicharo J. (2021). Discovery
of the propargyl radical (CH_2_CCH) in TMC-1: one of the
most abundant radicals ever found and a key species for cyclization
to benzene in cold dark clouds. Astron. Astrophys..

[36] Robertson C., Hyland R., Lacey A. J. D., Havens S., Habershon S. (2021). Identifying
Barrierless Mechanisms for Benzene Formation in the Interstellar Medium
Using Permutationally Invariant Reaction Discovery. J. Chem. Theory Comput..

[37] Murakami T., Takayanagi T. (2022). Interstellar Benzene Formation Mechanisms
via Acetylene
Cyclotrimerization Catalyzed by Fe^+^ Attached to Water Ice
Clusters: Quantum Chemistry Calculation Study. Molecules.

[38] Pentsak E. O., Murga M. S., Ananikov V. P. (2024). Role of
Acetylene in the Chemical
Evolution of Carbon Complexity. ACS Earth Space
Chem..

[39] Kocheril G. S., Zagorec-Marks C., Lewandowski H. J. (2025). Termination of bottom-up interstellar
aromatic ring formation at C_6_H_5_
^+^. Nat. Astron..

[40] Loison, J.-C. ; Rossi, C. ; Solem, N. ; Thissen, R. ; Romanzin, C. ; Alcaraz, C. ; Jacovella, U. Evidence for Phenylium Reactivity under Interstellar Relevant Conditions. arXiv 2506.13290v3, 2026; https://arxiv.org/abs/2506.13290v3, Accessed April 21, 2026.

[41] Abplanalp M. J., Kaiser R. I. (2020). Implications
for Extraterrestrial Hydrocarbon Chemistry:
Analysis of Acetylene (C_2_H_2_) and D_2_-acetylene (C_2_D_2_) Ices Exposed to Ionizing
Radiation via Ultraviolet-Visible Spectroscopy, Infrared Spectroscopy,
and Reflectron Time-of-flight Mass Spectrometry. Astrophys. J..

[42] Kleimeier N. F., Liu Y., Turner A. M., Young L. A., Chin C. H., Yang T., He X., Lo J. I., Cheng B. M., Kaiser R. I. (2022). Excited state photochemically
driven surface formation of benzene from acetylene ices on Pluto and
in the outer solar system. Phys. Chem. Chem.
Phys..

[43] Samarth P., Fedoseev G., Bulak M., Ioppolo S., Hornekær L., van Dishoeck E. F., Linnartz H., Chuang K. J. (2026). In situ characterization
of volatile and refractory hydrocarbons produced by UV photolysis
of interstellar C_2_H_2_ ice. Astron. Astrophys..

[44] Woon D. E. (2011). Ion-ice
astrochemistry: Barrierless low-energy deposition pathways to HCOOH,
CH_3_OH, and CO_2_ on icy grain mantles from precursor
cations. Astrophys. J..

[45] Cui W., Herbst E. (2024). Exploring the Role of Ion–Molecule Reactions
on Interstellar Icy Grain Surfaces. ACS Earth
Space Chem..

[46] Nakai Y., Sameera W. M. C., Furuya K., Hidaka H., Ishibashi A., Watanabe N. (2023). Methanol Formation through Reaction of Low-energy CH_3_
^+^ Ions with an Amorphous Solid Water Surface at
Low Temperature. Astrophys. J..

[47] De
Barros A. L. F., da Costa C. A. P., Murhej Y., Sreeja R., Doreste D. V., Da Silveira E. F., Bouduch P., Rothard H., Michielan M., Ascenzi D. (2026). Swift Heavy Ion-Induced
Chemistry of CH_3_CN Ices at 10 and 80 K. ACS Earth Space Chem..

[48] Matuszewski F. (2026). Ion induced formation
of complex organic nitrogen molecules in solid-phase
adenine. Icarus.

[49] Woon D. E. (2026). Quantum
Chemical Modeling of Astrophysical Ice Mantle Reactions of C^+^ with Ammonia, Cyanamide, Formamide, Hydroxylamine, and Carbamic
Acid. ACS Earth Space Chem..

[50] Albertini S., Gruber E., Zappa F., Krasnokutskiy S., Laimer F., Scheier P. (2022). Chemistry and physics
of dopants
embedded in helium droplets. Mass Spectrom.
Rev..

[51] Briant M., Mestdagh J.-M., Gaveau M.-A., Poisson L. (2022). Reaction dynamics within
a cluster environment. Phys. Chem. Chem. Phys..

[52] Christensen J. K., Petersen C. E., Albrechtsen S. H., Goudot J., Calvo F., Stapeldeldt H. (2026). Real-time
observation of the diffusion-limited formation
of a cation-molecule complex. Nature Commun..

[53] Mani D., de Tudela R. P., Schwan R., Pal N., Korning S., Forbert H., Redlich B., van der Meer A. F. G., Schwaab G., Marx D., Havenith M. (2019). Acid solvation versus
dissociation at “stardust conditions”: Reaction sequence
matters. Sci. Adv..

[54] Moon C. J., Erukala S., Feinberg A. J., Singh A., Choi M. Y., Vilesov A. F. (2023). Formation of the C_4_H_
*n*
_
^+^ (*n* = 2–5) ions upon ionization
of acetylene clusters in helium droplets. J.
Chem. Phys..

[55] Momoh P. O., Abrash S. A., Mabrouki R., El-Shall M. S. (2006). Polymerization of
ionized acetylene clusters into covalent bonded ions: Evidence for
the formation of benzene radical cation. J.
Am. Chem. Soc..

[56] Momoh P. O., El-Shall M. S. (2007). Stepwise hydration of ionized acetylene trimer. Further
evidence for the formation of benzene radical cation. Chem. Phys. Lett..

[57] Bergmeister S., Ganner L., Locher J., Zappa F., Scheier P., Gruber E. (2023). Spectroscopy of helium-tagged molecular
ionsDevelopment
of a novel experimental setup. Rev. Sci. Instrum..

[58] Feinberg A. J., Laimer F., Tanyag R. M. P., Senfftleben B., Ovcharenko Y., Dold S., Gatchell M., O’Connell-Lopez S. M., Erukala S., Saladrigas C. A. (2022). X-ray diffractive imaging
of highly ionized helium nanodroplets. Phys.
Rev. Research.

[59] Goode J. G., Hofstein J. D., Johnson P. M. (1997). The observation
of strong pseudo-JahnTeller
activity in the benzene cation B ^2^E_2*g*
_ state. J. Chem. Phys..

[60] Walter K., Weinkauf R., Boesl U., Schlag E. W. (1989). Spectroscopy of
the benzene cation: Resonance-enhanced multiphoton dissociation spectra
of the B­(E_2*g*
_)?X­(E_1*g*
_) transition. Chem. Phys. Lett..

[61] Frisch, M. J. Gaussian 16 Revision A.03; Gaussian Inc.: 2016.

[62] Smith M. A., Hawley M. (1995). Ion/molecule reaction
rate coefficients at translational
temperatures below 5 K: selected bimolecular reactions of C_2_H_2_
^+^ and NH_4_
^+^. Int. J. Mass Spectrom. Ion Process..

[63] Anicich, V. G. An index of the Literature for Bimolecular Gas Phase Cation-Molecule Reaction Kinetics; JPL Publications: 2003.

[64] Peverati R., Bera P. P., Lee T. J., Head-Gordon M. (2016). Insights into
hydrocarbon chain and aromatic ring formation in the interstellar
medium: computational study of the isomers of and and their formation
pathways. Astrophys. J..

[65] Chuang K. J., Fedoseev G., Scirè C., Baratta G. A., Jäger C., Henning T., Linnartz H., Palumbo M. E. (2021). Formation of complex
organic molecules in molecular clouds: acetaldehyde, vinyl alcohol,
ketene, and ethanol via the “energetic” processing of
C_2_H_2_ ice. Astron. Astrophys..

