# Supplementary material for: Cryogenic Bottom-up Formation of the Benzene Cation from Acetylene in Helium Nanodroplets
Source: J Am Chem Soc. 2026 May 2;148(18):18615–20. doi: 10.1021/jacs.6c04278 (PMC13185103; doi:10.1021/jacs.6c04278)
Supplement: Supplementary file 1 [file ja6c04278_si_001.pdf]

Supplementary Information: Cryogenic bottom-up formation of the benzene cation from acetylene in helium nanodroplets

Florian Foitzik,[†] Vincent Richardson,[‡] Colombe Maurice,[¶] Gabriel Schöpfer,[†] Milan Ončák,[†] and Elisabeth Gruber[*,†]

†*Department of Ion Physics and Applied Physics, University of Innsbruck, Innsbruck 6020, Austria*

‡*Department of Physics, University of Liverpool, Liverpool L69 7ZX, United Kingdom*

¶*ENS Paris-Saclay, Université Paris-Saclay, Gif-sur-Yvette 91190, France*

E-mail: E.Gruber@uibk.ac.at

Additional computational details and spectra computed with alternative methods

Calculation of vibrationally resolved spectra in the benzene radical cation ($C_6H_6^{\cdot+}$) is challenging as Jahn-Teller splitting reduces the symmetry point group from D_{6h} to D_{2h}. Previous studies[1,2] suggested two different minima in the ground electronic state that differ in the length of the C−C bonds. A B_{2g} minimum with four long and two short bonds, and a B_{3g} minimum with two long and four short bonds. However, almost all single-reference methods we tested (restricted MP2, unrestricted MP2, unrestricted CCSD, BMK, CAM-B3LYP, ωB97XD, BHandHLYP) predict the B_{3g} isomer to be a transition state, which hints towards possible need of multireference treatment. The only exception is restricted open-shell CCSD that predicts the B_{3g} isomer to be a minimum. At the MRCI(3,4)/def2-TZVP level, two minima (B_{2g} and B_{3g}) were obtained. However, the difference between both stationary points lies consistently below 0.1 eV with all methods. Therefore, the potential energy surface along the dimension connecting both minima is presumably floppy, and hence, harmonic approximation is most probably not appropriate.

In the D_2 excited state (also denoted as B state), further symmetry lowering to C_{2v} is predicted by all used DFT functionals within the time-dependent DFT formalism, as well as Equation of Motion CCSD (EOM-CCSD). The only exception is TD-BMK which predicts D_{2h} symmetry to be preserved upon excitation, and thus no spectrum is obtained with this method as the excitation is symmetry forbidden. The C_{2v} structure of the D_2 state has two long and four short C−C bonds. Hydrogen atoms that are connected to C atoms that form the two two short C−C bonds lie above the ring plane, while the other four hydrogen atoms lie below the plane. The energy difference between C_{2v} and D_{2h} stationary points is, however, minimal, pointing again towards the shallowness of the potential energy surface as well as possible multireference issues. Within the harmonic approximation, we still consider that from the B_{2g} minimum in the ground state, three D_2 minima can be reached. The three

D_2 minima are all equivalent, but differ by the relative position of short C−C bonds with respect to the ground-state structure. The resulting spectra are shown in Figure S1.

MRCI, CCSD and MP2 calculations were performed in Molpro 2012.1.[3]

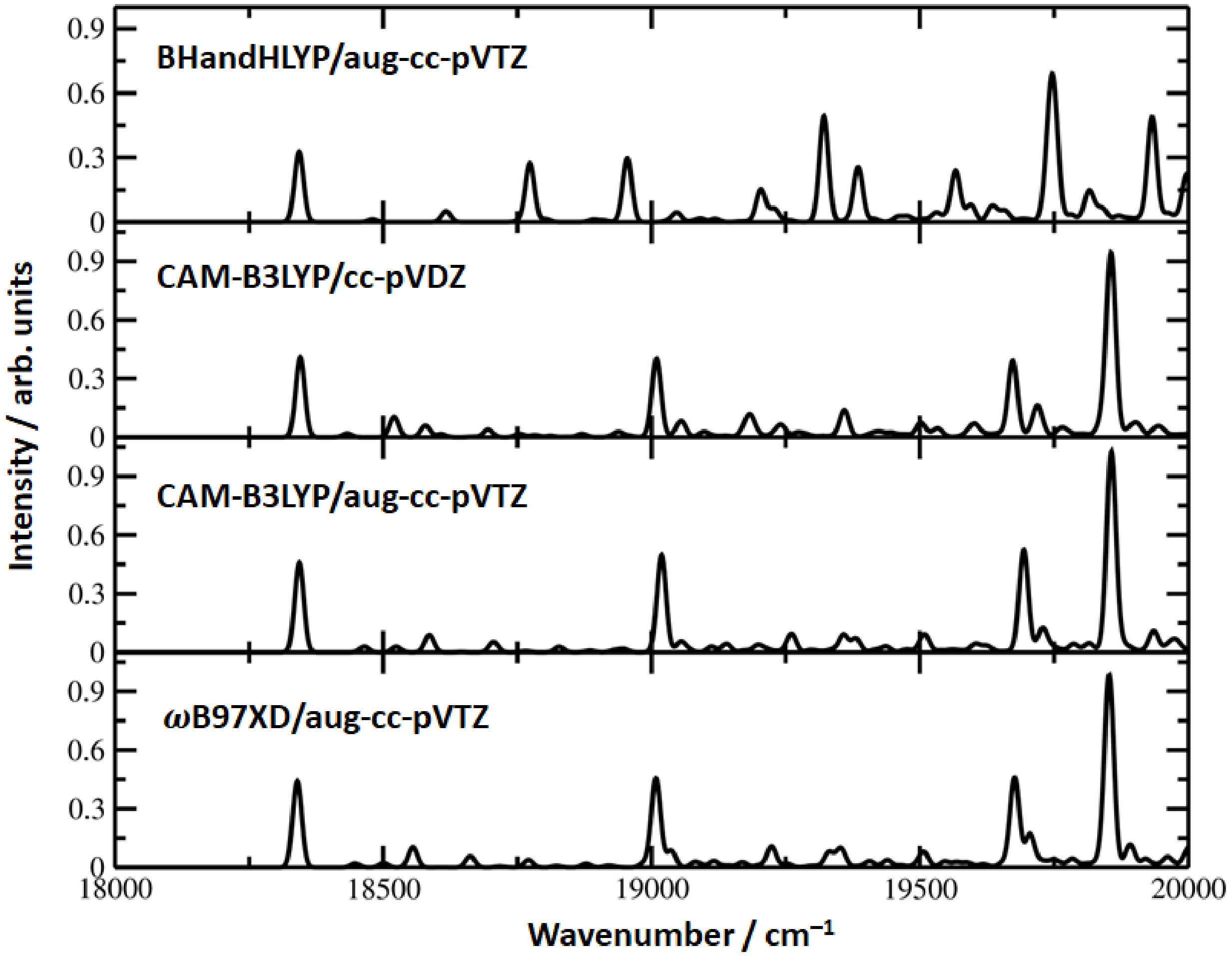

Figure S1: Franck-Condon spectra calculated using various levels of theory. The following shifts were applied to match the first experimental transition: −4200 cm^{-1} for BHandHLYP/aug-cc-pVTZ, −510 cm^{-1} for CAM-B3LYP/cc-pVDZ, −260 cm^{-1} for CAM-B3LYP/aug-cc-pVTZ, −760 cm^{-1} for ωB97XD/aug-cc-pVTZ.

Energy, structure, and vibrational frequencies of optimized structures

UCCSD/aug-cc-pVDZ, B2g optimum
Electronic energy (in Hartree):
-231.24392079
Structure (in Angstrom):
C 0.0000000000 1.3918444101 0.0000000000
C 1.2587535344 0.6904733696 0.0000000000
C 1.2587535344 -0.6904733696 0.0000000000
C 0.0000000000 -1.3918444101 0.0000000000
C -1.2587535344 -0.6904733696 0.0000000000
C -1.2587535344 0.6904733696 0.0000000000
H 2.1908020554 -1.2615369366 0.0000000000
H 0.0000000000 -2.4874589302 0.0000000000
H -2.1908020554 -1.2615369366 0.0000000000
H -2.1908020554 1.2615369366 0.0000000000
H 0.0000000000 2.4874589302 0.0000000000
H 2.1908020554 1.2615369366 0.0000000000
Frequencies (in cm-1):
280.88 285.13 324.15 394.81 584.88
682.97 797.76 844.14 954.99 955.14
974.27 975.62 980.46 981.39 987.72
1064.86 1187.03 1202.61 1351.03 1364.55
1423.80 1432.13 1536.74 1685.59 3213.53
3216.41 3228.26 3231.74 3243.15 3246.61

UCCSD/aug-cc-pVDZ, B3g optimum
Electronic energy (in Hartree):
-231.24359759
Structure (in Angstrom):
C 0.0000000000 1.4504991686 0.0000000000
C 1.2012207149 0.7309645758 0.0000000000
C 1.2012207149 -0.7309645758 0.0000000000
C 0.0000000000 -1.4504991686 0.0000000000
C -1.2012207149 -0.7309645758 0.0000000000
C -1.2012207149 0.7309645758 0.0000000000
H 2.1639891463 -1.2521614239 0.0000000000
H 0.0000000000 -2.5426947581 0.0000000000
H -2.1639891463 -1.2521614239 0.0000000000
H -2.1639891463 1.2521614239 0.0000000000
H 0.0000000000 2.5426947581 0.0000000000

H 2.1639891463 1.2521614239 0.0000000000
Frequencies (in cm-1):
i506.68 260.45 280.68 343.16 578.43
708.35 790.85 852.05 862.88 891.27
946.63 963.27 963.33 967.44 978.00
1079.48 1152.39 1156.83 1306.91 1339.74
1410.80 1439.56 1523.01 1591.09 3203.81
3204.48 3220.34 3224.36 3240.47 3241.65

RCCSD/aug-cc-pVDZ, B2g optimum
Electronic energy (in Hartree):
-231.24275670
Structure (in Angstrom):
C 0.0000000000 1.3913065506 0.0000000000
C 1.2585776921 0.6904636267 0.0000000000
C 1.2585776921 -0.6904636267 0.0000000000
C 0.0000000000 -1.3913065506 0.0000000000
C -1.2585776921 -0.6904636267 0.0000000000
C -1.2585776921 0.6904636267 0.0000000000
H 2.1904971459 -1.2616999145 0.0000000000
H 0.0000000000 -2.4869237543 0.0000000000
H -2.1904971459 -1.2616999145 0.0000000000
H -2.1904971459 1.2616999145 0.0000000000
H 0.0000000000 2.4869237543 0.0000000000
H 2.1904971459 1.2616999145 0.0000000000
Frequencies (in cm-1):
226.95 248.67 291.49 357.30 580.41
714.76 763.47 824.79 924.39 951.43
954.75 966.35 977.59 978.17 1028.88
1069.26 1150.23 1165.18 1335.09 1347.71
1422.93 1473.51 1544.49 1682.42 3207.29
3213.80 3215.16 3221.88 3236.77 3243.45

RCCSD/aug-cc-pVDZ, B3g optimum
Electronic energy (in Hartree):
-231.24261997
Structure (in Angstrom):
C 0.0000000000 1.4503826982 0.0000000000
C 1.2009323051 0.7309448767 0.0000000000
C 1.2009323051 -0.7309448767 0.0000000000
C 0.0000000000 -1.4503826982 0.0000000000
C -1.2009323051 -0.7309448767 0.0000000000
C -1.2009323051 0.7309448767 0.0000000000
H 2.1637214515 -1.2520739535 0.0000000000
H 0.0000000000 -2.5425833914 0.0000000000

H -2.1637214515 -1.2520739535 0.0000000000
H -2.1637214515 1.2520739535 0.0000000000
H 0.0000000000 2.5425833914 0.0000000000
H 2.1637214515 1.2520739535 0.0000000000
Frequencies (in cm-1):
276.53 292.84 308.81 353.15 581.16
702.80 800.15 870.90 901.54 955.16
966.18 971.65 973.43 979.39 1005.66
1076.03 1162.31 1179.37 1345.16 1370.81
1426.85 1460.91 1519.54 1594.55 3210.46
3212.39 3222.33 3227.09 3242.30 3243.68

UMP2/aug-cc-pVDZ, B3g optimum
Electronic energy (in Hartree):
-231.19024736
Structure (in Angstrom):
C 0.0000000000 1.4466733682 0.0000000000
C 1.1976877770 0.7298988223 0.0000000000
C 1.1976877770 -0.7298988223 0.0000000000
C 0.0000000000 -1.4466733682 0.0000000000
C -1.1976877770 -0.7298988223 0.0000000000
C -1.1976877770 0.7298988223 0.0000000000
H 2.1593863243 -1.2514557461 0.0000000000
H 0.0000000000 -2.5383282999 0.0000000000
H -2.1593863243 -1.2514557461 0.0000000000
H -2.1593863243 1.2514557461 0.0000000000
H 0.0000000000 2.5383282999 0.0000000000
H 2.1593863243 1.2514557461 0.0000000000
Frequencies (in cm-1):
i710.29 281.49 302.75 355.26 579.96
701.00 806.59 809.91 878.26 933.78
980.97 985.06 991.37 993.16 1039.93
1175.07 1175.79 1189.53 1303.30 1339.45
1438.32 1567.51 1588.82 1631.95 3230.52
3233.90 3239.32 3244.83 3260.37 3260.45

RMP2/aug-cc-pVDZ, B2g optimum
Electronic energy (in Hartree):
-231.20205198
Structure (in Angstrom):
C 0.0000000000 1.3949647843 0.0000000000
C 1.2559322648 0.6932870551 0.0000000000
C 1.2559322648 -0.6932870551 0.0000000000
C 0.0000000000 -1.3949647843 0.0000000000
C -1.2559322648 -0.6932870551 0.0000000000

C -1.2559322648 0.6932870551 0.0000000000
H 2.1904791988 -1.2596710063 0.0000000000
H 0.0000000000 -2.4899798427 0.0000000000
H -2.1904791988 -1.2596710063 0.0000000000
H -2.1904791988 1.2596710063 0.0000000000
H 0.0000000000 2.4899798427 0.0000000000
H 2.1904791988 1.2596710063 0.0000000000
Frequencies (in cm-1):
283.10 324.86 382.76 406.71 613.51
672.52 786.19 816.80 952.14 958.56
965.31 971.80 983.20 1014.13 1061.33
1186.58 1204.33 1227.10 1339.06 1369.99
1474.64 1549.34 1655.22 2100.85 3229.97
3241.98 3244.01 3254.54 3256.40 3290.77

RMP2/aug-cc-pVDZ, B3g optimum
Electronic energy (in Hartree):
-231.20149049
Structure (in Angstrom):
C 0.0000000000 1.4478368484 0.0000000000
C 1.2043755897 0.7293269510 0.0000000000
C 1.2043755897 -0.7293269510 0.0000000000
C 0.0000000000 -1.4478368484 0.0000000000
C -1.2043755897 -0.7293269510 0.0000000000
C -1.2043755897 0.7293269510 0.0000000000
H 2.1646522762 -1.2541723232 0.0000000000
H 0.0000000000 -2.5397825326 0.0000000000
H -2.1646522762 -1.2541723232 0.0000000000
H -2.1646522762 1.2541723232 0.0000000000
H 0.0000000000 2.5397825326 0.0000000000
H 2.1646522762 1.2541723232 0.0000000000
Frequencies (in cm-1):
i837.55 296.31 310.60 353.73 577.48
675.20 784.26 808.41 868.11 915.08
959.19 962.27 964.05 972.61 975.69
1074.11 1186.79 1196.69 1325.30 1338.95
1406.14 1495.54 1571.40 2083.26 3226.60
3232.14 3236.74 3242.49 3257.14 3258.47

EOMCCSD/cc-pVDZ, D2 optimum
Electronic energy (in Hartree):
-231.20001493
Structure (in Angstrom):
C 1.210996 -0.770787 -0.014624
C 1.210996 0.621555 0.025049

C 0.079747 1.433333 -0.014611
C -1.210996 0.770787 -0.014624
C -1.210996 -0.621555 0.025049
C -0.079747 -1.433333 -0.014611
H 0.089017 2.524171 -0.131281
H -2.102665 1.399225 -0.131434
H -2.177229 -1.117523 0.287836
H -0.089017 -2.524171 -0.131281
H 2.102665 -1.399225 -0.131434
H 2.177229 1.117523 0.287836
Frequencies (in cm-1):
170.3599 365.9740 411.2827
562.3235 598.0622 656.6774
734.9374 743.5475 791.7715
880.2491 884.1335 965.5300
968.3266 1022.5099 1030.7267
1042.3806 1126.1886 1133.7926
1151.6752 1260.7801 1357.7530
1400.3556 1409.8680 1448.4197
2921.4490 2961.0985 3171.7327
3199.7016 3225.2338 3226.1033

EOMCCSD/cc-pVDZ, D2 optimum, D2h symmetry
Electronic energy (in Hartree):
-231.21396356
Structure (in Angstrom):
C -0.000000 0.000000 1.440224
C 0.000000 1.244058 0.723753
C -0.000000 1.244058 -0.723753
C 0.000000 -0.000000 -1.440224
C -0.000000 -1.244058 -0.723753
C 0.000000 -1.244058 0.723753
H 0.000000 2.192751 -1.269854
H 0.000000 -0.000000 -2.535043
H -0.000000 -2.192751 -1.269854
H -0.000000 -2.192751 1.269854
H 0.000000 0.000000 2.535043
H -0.000000 2.192751 1.269854
Frequencies (in cm-1):
i1170.8244 -942.1309 473.7317
481.0762 556.5787 595.9801
604.7386 654.8335 679.8444
682.4947 896.1598 963.4527
1006.6060 1069.2615 1088.2545
1173.7166 1178.1812 1230.2047

1359.4950 1394.3032 1529.3415
1562.0893 1588.1692 1925.7639
3239.5884 3243.9237 3245.4455
3256.6176 3258.4581 3262.3369

BMK/cc-pVDZ, B2g optimum
Electronic energy (in Hartree):
-231.754626450
Structure (in Angstrom):
C -0.000000 0.000000 1.388915
C -0.000000 1.256460 0.688013
C 0.000000 1.256460 -0.688013
C 0.000000 0.000000 -1.388915
C -0.000000 -1.256460 -0.688013
C -0.000000 -1.256460 0.688013
H 0.000000 2.190106 -1.258519
H 0.000000 0.000000 -2.485477
H -0.000000 -2.190106 -1.258519
H -0.000000 -2.190106 1.258519
H -0.000000 -0.000000 2.485477
H -0.000000 2.190106 1.258519
Frequencies (in cm-1):
289.1969 343.3155 361.8899
425.3736 594.1665 685.8827
808.0528 906.3609 961.7714
979.3706 996.1361 1000.4014
1010.8182 1028.5079 1036.5720
1066.3929 1188.5209 1207.1075
1369.1503 1378.5480 1414.7191
1440.9826 1539.4006 1681.2891
3211.9199 3215.2435 3231.0956
3233.5954 3243.7818 3246.4597

BMK/cc-pVDZ, B3g optimum
Electronic energy (in Hartree):
-231.754103921
Structure (in Angstrom):
C 0.000000 0.000000 1.447191
C -0.000000 1.198337 0.730416
C 0.000000 1.198337 -0.730416
C 0.000000 0.000000 -1.447191
C -0.000000 -1.198337 -0.730416
C -0.000000 -1.198337 0.730416
H -0.000000 2.161495 -1.253106
H -0.000000 0.000000 -2.540606

H -0.000000 -2.161495 -1.253106
H -0.000000 -2.161495 1.253106
H -0.000000 -0.000000 2.540606
H -0.000000 2.161495 1.253106
Frequencies (in cm-1):
i605.5801 306.1872 330.1498
401.5783 594.5200 685.4936
839.7441 853.7780 885.5634
950.2083 977.7776 997.1606
1018.5334 1020.4450 1024.9010
1078.4644 1186.9077 1196.5999
1326.3899 1368.0736 1405.9716
1453.0563 1524.3520 1591.5157
3215.1602 3220.6100 3224.0787
3228.3263 3240.4883 3243.1549

BMK/cc-pVDZ, B3g optimum
Electronic energy (in Hartree):
-231.735902225
Structure (in Angstrom):
C 1.233357 0.724068 0.000000
C 0.000000 1.332465 -0.000000
C -1.233357 0.724068 0.000000
C -1.233357 -0.724068 0.000000
C 0.000000 -1.332465 -0.000000
C 1.233357 -0.724068 0.000000
H -2.214320 1.216294 -0.000000
H -2.214320 -1.216294 -0.000000
H 0.000000 -2.462516 -0.000001
H 2.214320 -1.216294 -0.000000
H 2.214320 1.216294 -0.000000
H -0.000000 2.462516 -0.000001
Frequencies (in cm-1):
102.6362 195.3270 422.4043
553.3000 568.7093 660.0709
712.3094 713.9586 732.1120
878.1341 899.6145 942.5675
945.0264 1001.4587 1019.2489
1033.6282 1079.6796 1150.7135
1217.6696 1265.5267 1359.9359
1376.2809 1504.8627 2690.3707
2874.8430 3110.1887 3162.6256
3195.7035 3208.3700 3318.3064

BMK/cc-pVDZ, D2 optimum, D2h

Electronic energy (in Hartree):
-231.735902133
Structure (in Angstrom):
C 0.000000 1.233356 0.724069
C 0.000000 -0.000000 1.332468
C -0.000000 -1.233356 0.724069
C -0.000000 -1.233356 -0.724069
C 0.000000 -0.000000 -1.332468
C 0.000000 1.233356 -0.724069
H 0.000000 -2.214322 1.216288
H 0.000000 -2.214322 -1.216288
H -0.000000 0.000000 -2.462518
H 0.000000 2.214322 -1.216288
H 0.000000 2.214322 1.216288
H -0.000000 0.000000 2.462518
Frequencies (in cm-1):
102.7134 195.3285 422.4044
553.3082 568.7082 660.0695
712.3079 713.9576 732.0979
878.1300 899.6140 942.5618
945.0251 1001.4564 1019.2470
1033.6288 1079.6800 1150.7154
1217.6683 1265.5315 1359.9367
1376.2814 1504.8594 2690.3843
2874.8520 3110.1815 3162.6287
3195.7065 3208.3728 3317.9580

BMK/aug-cc-pVTZ, B2G optimum
Electronic energy (in Hartree):
-231.819163589
Structure (in Angstrom):
C -0.000000 0.000000 1.379415
C 0.000000 1.249285 0.682456
C 0.000000 1.249285 -0.682456
C 0.000000 -0.000000 -1.379415
C -0.000000 -1.249285 -0.682456
C -0.000000 -1.249285 0.682456
H 0.000000 2.172383 -1.247394
H 0.000000 0.000000 -2.463823
H -0.000000 -2.172383 -1.247394
H -0.000000 -2.172383 1.247394
H -0.000000 0.000000 2.463823
H -0.000000 2.172383 1.247394
Frequencies (in cm-1):
252.8390 324.0761 357.5926

359.9647 600.5774 687.6653
811.8233 868.1763 958.0388
971.7615 992.1196 995.5847
1008.1320 1017.1069 1019.5984
1066.3505 1204.2730 1223.6974
1384.9957 1390.7594 1400.2646
1461.9072 1548.2430 1672.1472
3186.1798 3190.4986 3200.4886
3203.8283 3212.5095 3215.4777

BMK/aug-cc-pVTZ, B3G optimum
Electronic energy (in Hartree):
-231.818629428
Structure (in Angstrom):
C 0.000000 0.000000 1.438308
C -0.000000 1.189540 0.726947
C 0.000000 1.189540 -0.726947
C 0.000000 0.000000 -1.438308
C -0.000000 -1.189540 -0.726947
C -0.000000 -1.189540 0.726947
H -0.000000 2.142466 -1.243010
H 0.000000 -0.000000 -2.519765
H -0.000000 -2.142466 -1.243010
H -0.000000 -2.142466 1.243010
H -0.000000 -0.000000 2.519765
H -0.000000 2.142466 1.243010
Frequencies (in cm-1):
i569.9645 276.3451 308.3982
337.8314 601.0536 686.4261
829.9121 847.7704 888.9424
943.8921 971.6115 979.6566
1006.5151 1007.0905 1015.1845
1080.4379 1200.9512 1206.7053
1346.9595 1378.7424 1398.4999
1461.4563 1534.6459 1578.8256
3187.6958 3193.0820 3197.3379
3200.6904 3213.5745 3216.0046

BMK/aug-cc-pVTZ, D2 optimum, C2v
Electronic energy (in Hartree):
-231.799895993
Structure (in Angstrom):
6 1.224343 0.720544 0.000154
6 0.000000 1.322504 -0.000179
6 -1.224343 0.720544 0.000154

6 -1.224343 -0.720544 0.000154
6 0.000000 -1.322504 -0.000179
6 1.224343 -0.720544 0.000154
1 -2.194533 1.208079 0.000707
1 -2.194533 -1.208079 0.000707
1 0.000000 -2.441971 -0.002188
1 2.194533 -1.208079 0.000707
1 2.194533 1.208079 0.000707
1 -0.000000 2.441971 -0.002188
Frequencies (in cm-1):
82.9536 148.0457 408.5761
556.7533 559.8102 642.9351
664.4180 682.0271 712.2895
870.7479 891.3627 929.9766
941.7358 989.7031 1028.4937
1040.2773 1087.0124 1158.1244
1245.3758 1293.1749 1333.1484
1382.4214 1486.7470 2662.2429
2794.3324 2847.0092 3099.5735
3133.7652 3167.6552 3180.3613

BMK/aug-cc-pVTZ, D2 optimum, D2h
Electronic energy (in Hartree):
-231.799896603
Structure (in Angstrom):
6 0.000000 1.224343 0.720544
6 0.000000 0.000000 1.322505
6 -0.000000 -1.224343 0.720544
6 -0.000000 -1.224343 -0.720544
6 0.000000 0.000000 -1.322505
6 0.000000 1.224343 -0.720544
1 0.000000 -2.194533 1.208079
1 0.000000 -2.194533 -1.208079
1 0.000000 -0.000000 -2.441974
1 0.000000 2.194533 -1.208079
1 0.000000 2.194533 1.208079
1 -0.000000 -0.000000 2.441974
Frequencies (in cm-1):
83.6230 147.4744 403.4981
556.5526 559.5933 641.0453
664.3182 681.9825 712.8508
868.5929 891.3701 932.8815
941.7278 989.6848 1028.0576
1040.2586 1087.0140 1158.1213
1245.4136 1292.3116 1333.0830

1382.3884 1486.6364 2662.2520
2794.1200 2847.0102 3099.5884
3133.7818 3167.6713 3180.3810

CAM-B3LYP/cc-pVDZ, B2G optimum
Electronic energy (in Hartree):
-231.785432252
Structure (in Angstrom):
C -0.000000 0.000000 1.379420
C -0.000000 1.247123 0.683448
C 0.000000 1.247123 -0.683448
C 0.000000 0.000000 -1.379420
C -0.000000 -1.247123 -0.683448
C -0.000000 -1.247123 0.683448
H 0.000000 2.178126 -1.251881
H 0.000000 0.000000 -2.472790
H -0.000000 -2.178126 -1.251881
H -0.000000 -2.178126 1.251881
H -0.000000 -0.000000 2.472790
H -0.000000 2.178126 1.251881
Frequencies (in cm-1):
260.7012 296.1142 349.4000
441.6989 603.1052 690.6223
813.6405 919.7931 975.1449
977.6328 999.1652 1012.3096
1022.7406 1041.8279 1054.2483
1080.7155 1192.8255 1212.2946
1373.0382 1379.8081 1433.8421
1451.5800 1563.3550 1717.0325
3216.3439 3219.9904 3233.5464
3236.4342 3245.9587 3248.7448

CAM-B3LYP/cc-pVDZ, B3G optimum
Electronic energy (in Hartree):
-231.785224824
Structure (in Angstrom):
C -0.000000 0.000000 1.436400
C -0.000000 1.190047 0.724805
C 0.000000 1.190047 -0.724805
C 0.000000 -0.000000 -1.436400
C -0.000000 -1.190047 -0.724805
C -0.000000 -1.190047 0.724805
H -0.000000 2.149950 -1.246171
H 0.000000 0.000000 -2.526323
H -0.000000 -2.149950 -1.246171

H -0.000000 -2.149950 1.246171
H -0.000000 0.000000 2.526323
H -0.000000 2.149950 1.246171
Frequencies (in cm-1):
i321.8423 310.6560 336.3887
417.9919 603.5374 690.2611
862.3688 893.1957 908.9651
967.2662 998.5455 1008.2319
1031.0994 1032.4685 1045.1645
1092.9226 1192.1712 1203.8097
1346.4267 1379.4731 1422.4114
1480.3666 1541.6314 1618.4921
3220.2483 3225.2781 3229.9269
3233.2032 3248.4363 3250.6035

CAM-B3LYP/cc-pVDZ, D2 optimum, C2v
Electronic energy (in Hartree):
-231.836111270
Structure (in Angstrom):
C 1.217504 0.714729 -0.005035
C 0.000000 1.317108 0.008415
C -1.217504 0.714729 -0.005035
C -1.217504 -0.714729 -0.005035
C -0.000000 -1.317108 0.008415
C 1.217504 -0.714729 -0.005035
H -2.182759 1.203696 -0.051119
H -2.182759 -1.203696 -0.051119
H 0.000000 -2.427047 0.112167
H 2.182759 -1.203696 -0.051119
H 2.182759 1.203696 -0.051119
H 0.000000 2.427047 0.112167
Frequencies (in cm-1):
121.1253 180.6161 412.2425
546.2643 587.1988 674.8813
710.6395 738.5060 808.3925
870.8361 901.9109 959.8272
966.2399 1013.9895 1022.5730
1053.8894 1111.2626 1166.6426
1256.1436 1258.2198 1378.5291
1411.4561 1512.7753 1899.0203
2704.8582 2871.5501 3123.2759
3160.4988 3193.4537 3203.6891

CAM-B3LYP/cc-pVDZ, D2 optimum, D2h
Electronic energy (in Hartree):

-231.766955452
Structure (in Angstrom):
C 0.000000 1.225256 0.717879
C 0.000000 0.000000 1.321677
C -0.000000 -1.225256 0.717879
C -0.000000 -1.225256 -0.717879
C 0.000000 0.000000 -1.321677
C 0.000000 1.225256 -0.717879
H 0.000000 -2.201727 1.210313
H 0.000000 -2.201727 -1.210313
H -0.000000 -0.000000 -2.447756
H 0.000000 2.201727 -1.210313
H 0.000000 2.201727 1.210313
H -0.000000 -0.000000 2.447756
Frequencies (in cm-1):
i184.8666 74.9466 413.0597
532.6839 558.9428 669.9299
698.6675 725.2410 780.3230
867.0944 906.3149 946.1633
957.9873 1023.3756 1023.4028
1048.4761 1087.8244 1157.5564
1226.7979 1246.4318 1394.5327
1395.6194 1535.4550 1762.3285
2691.7725 2879.2936 3130.7396
3169.8963 3203.1903 3214.8867

CAM-B3LYP/aug-cc-pVTZ, B2G optimum
Electronic energy (in Hartree):
-231.855199142
Structure (in Angstrom):
C 0.000000 0.000000 1.371085
C -0.000000 1.240527 0.678818
C 0.000000 1.240527 -0.678818
C 0.000000 -0.000000 -1.371085
C -0.000000 -1.240527 -0.678818
C -0.000000 -1.240527 0.678818
H -0.000000 2.162195 -1.241875
H -0.000000 -0.000000 -2.453365
H -0.000000 -2.162195 -1.241875
H -0.000000 -2.162195 1.241875
H -0.000000 0.000000 2.453365
H -0.000000 2.162195 1.241875
Frequencies (in cm-1):
255.7449 282.5503 343.0614
423.5268 608.3920 695.1104

816.7868 917.6880 975.3761
977.8726 996.3311 1020.6919
1027.2700 1043.5329 1046.9008
1083.3867 1208.2755 1227.3371
1388.0870 1405.5483 1416.5701
1470.9622 1570.0948 1709.6498
3206.4366 3210.1246 3222.5398
3225.4795 3234.2830 3237.1807

CAM-B3LYP/aug-cc-pVTZ, B3G optimum
Electronic energy (in Hartree):
-231.854992374
Structure (in Angstrom):
C -0.000000 0.000000 1.428539
C -0.000000 1.182602 0.721419
C 0.000000 1.182602 -0.721419
C 0.000000 0.000000 -1.428539
C -0.000000 -1.182602 -0.721419
C -0.000000 -1.182602 0.721419
H -0.000000 2.133125 -1.237225
H 0.000000 0.000000 -2.507884
H -0.000000 -2.133125 -1.237225
H -0.000000 -2.133125 1.237225
H -0.000000 -0.000000 2.507884
H -0.000000 2.133125 1.237225
Frequencies (in cm-1):
i314.2182 299.1622 329.3978
399.2068 609.5567 695.2884
862.5096 898.2294 913.2931
965.8144 995.4202 1023.9829
1029.6971 1034.1420 1034.9030
1097.9024 1207.4118 1215.2368
1365.2534 1405.6169 1414.7560
1483.7844 1553.4082 1612.2860
3209.2868 3214.1421 3218.8111
3222.0084 3235.8047 3238.1152

CAM-B3LYP/aug-cc-pVTZ, D2 optimum, C2v
Electronic energy (in Hartree):
-231.766314510
Structure (in Angstrom):
C 1.225842 0.717855 -0.007286
C 0.000000 1.327650 0.012198
C -1.225842 0.717855 -0.007286
C -1.225842 -0.717855 -0.007286

C -0.000000 -1.327650 0.012198
C 1.225842 -0.717855 -0.007286
H -2.198087 1.214300 -0.071490
H -2.198087 -1.214300 -0.071490
H 0.000000 -2.441809 0.157232
H 2.198087 -1.214300 -0.071490
H 2.198087 1.214300 -0.071490
H 0.000000 2.441809 0.157232
Frequencies (in cm-1):
87.5009 233.2358 416.2176
540.9001 593.7954 663.7000
710.4980 751.4010 854.3140
857.1029 902.5991 961.3542
971.1654 1007.6301 1013.9944
1046.1822 1112.1140 1156.1394
1216.5730 1237.3468 1395.2405
1414.5183 1510.9183 2223.5321
2738.1298 2885.9895 3137.2509
3173.6318 3205.0785 3214.0974

CAM-B3LYP/aug-cc-pVTZ, D2 optimum, D2h
Electronic energy (in Hartree):
-231.836448129
Structure (in Angstrom):
6 -0.000000 1.217199 0.714774
6 -0.000000 0.000000 1.314163
6 -0.000000 -1.217199 0.714774
6 -0.000000 -1.217199 -0.714774
6 0.000000 0.000000 -1.314163
6 0.000000 1.217199 -0.714774
1 -0.000000 -2.184607 1.201761
1 0.000000 -2.184607 -1.201761
1 0.000000 -0.000000 -2.430235
1 0.000000 2.184607 -1.201761
1 0.000000 2.184607 1.201761
1 -0.000000 -0.000000 2.430235
Frequencies (in cm-1):
i140.6219 119.3074 407.7536
543.2621 565.0430 678.1688
703.8233 709.4222 766.3636
880.7273 895.9809 945.4755
957.3747 1017.8554 1034.5090
1055.2282 1097.7157 1167.1088
1250.7282 1273.7374 1378.2506
1399.8019 1525.1653 1719.0572

2681.9460 2868.4446 3120.2153
3158.5421 3192.5136 3204.0648

wB97XD/cc-pVDZ, B2G optimum
Electronic energy (in Hartree):
-231.847416601
Structure (in Angstrom):
C 0.000000 0.000000 1.380889
C 0.000000 1.248514 0.684294
C 0.000000 1.248514 -0.684294
C 0.000000 0.000000 -1.380889
C -0.000000 -1.248514 -0.684294
C -0.000000 -1.248514 0.684294
H -0.000000 2.179242 -1.252498
H -0.000000 0.000000 -2.473846
H -0.000000 -2.179242 -1.252498
H -0.000000 -2.179242 1.252498
H -0.000000 -0.000000 2.473846
H -0.000000 2.179242 1.252498
Frequencies (in cm-1):
291.7127 344.5043 354.2495
435.2012 600.9146 687.1400
808.4615 912.5261 974.6014
993.7793 1004.7582 1008.3707
1015.1692 1033.7296 1041.8268
1078.7459 1198.7385 1216.3213
1375.5512 1385.5128 1432.4072
1449.1476 1558.7286 1711.3187
3218.7101 3221.8711 3239.7150
3241.9230 3252.4141 3254.9368

wB97XD/cc-pVDZ, B3G optimum
Electronic energy (in Hartree):
-231.846946890
Structure (in Angstrom):
C -0.000000 0.000000 1.438017
C 0.000000 1.191512 0.725589
C 0.000000 1.191512 -0.725589
C 0.000000 -0.000000 -1.438017
C -0.000000 -1.191512 -0.725589
C -0.000000 -1.191512 0.725589
H 0.000000 2.151131 -1.246861
H 0.000000 0.000000 -2.527682
H -0.000000 -2.151131 -1.246861
H -0.000000 -2.151131 1.246861

H -0.000000 0.000000 2.527682
H -0.000000 2.151131 1.246861
Frequencies (in cm-1):
i573.8968 306.5181 331.2062
408.3968 601.4991 686.2901
856.2776 857.6191 887.5366
964.9367 993.6071 1004.8732
1023.1486 1023.7602 1030.7923
1092.0524 1197.4226 1207.3221
1334.1793 1375.2176 1420.4840
1476.0928 1537.4649 1613.0689
3223.8230 3229.0214 3233.0970
3236.8402 3250.8248 3253.1800

wB97XD/cc-pVDZ, D2 optimum, C2v
Electronic energy (in Hartree):
-231.828415186
Structure (in Angstrom):
C 1.226892 0.718434 -0.004585
C 0.000000 1.325307 0.007598
C -1.226892 0.718434 -0.004585
C -1.226892 -0.718434 -0.004585
C -0.000000 -1.325307 0.007598
C 1.226892 -0.718434 -0.004585
H -2.201129 1.213117 -0.047153
H -2.201129 -1.213117 -0.047153
H 0.000000 -2.445941 0.103733
H 2.201129 -1.213117 -0.047153
H 2.201129 1.213117 -0.047153
H 0.000000 2.445941 0.103733
Frequencies (in cm-1):
85.1108 148.5078 398.4106
527.2188 571.3810 662.3946
693.3802 726.0757 821.7866
873.3082 889.9778 951.3951
962.1477 1007.0175 1014.5562
1043.2530 1102.6966 1159.1967
1227.2055 1231.8673 1399.5489
1402.5130 1520.5046 2713.0275
2882.5069 3131.0903 3174.7244
3206.9245 3216.6526 4168.9201

wB97XD/cc-pVDZ, D2 optimum, D2h
Electronic energy (in Hartree):
-231.828726618

Structure (in Angstrom):
C 0.000000 1.226658 0.718453
C 0.000000 -0.000000 1.322820
C -0.000000 -1.226658 0.718453
C -0.000000 -1.226658 -0.718453
C 0.000000 -0.000000 -1.322820
C 0.000000 1.226658 -0.718453
H 0.000000 -2.202672 1.211521
H 0.000000 -2.202672 -1.211521
H -0.000000 0.000000 -2.448601
H 0.000000 2.202672 -1.211521
H 0.000000 2.202672 1.211521
H -0.000000 0.000000 2.448601
Frequencies (in cm-1):
i135.1743 79.1086 396.0215
524.1057 556.5722 666.2653
682.9055 713.4623 788.8589
887.4920 887.7891 930.6002
959.8347 1012.4687 1018.1176
1043.4799 1089.8236 1159.1485
1222.1067 1245.1717 1389.6398
1402.6742 1530.4546 2694.2862
2880.5944 3124.9908 3172.6356
3205.5849 3216.4800 3743.3151

wB97XD/aug-cc-pVTZ, B2G optimum
Electronic energy (in Hartree):
-231.909113219
Structure (in Angstrom):
C -0.000000 0.000000 1.373266
C -0.000000 1.242288 0.679956
C 0.000000 1.242288 -0.679956
C 0.000000 -0.000000 -1.373266
C -0.000000 -1.242288 -0.679956
C -0.000000 -1.242288 0.679956
H 0.000000 2.164138 -1.243214
H 0.000000 0.000000 -2.455773
H -0.000000 -2.164138 -1.243214
H -0.000000 -2.164138 1.243214
H -0.000000 0.000000 2.455773
H -0.000000 2.164138 1.243214
Frequencies (in cm-1):
277.0805 337.6005 361.4104
413.0290 605.3602 693.2024
812.6688 908.2212 974.3182

991.3843 1007.2552 1013.7430
1021.4351 1033.6140 1034.6714
1081.1955 1210.6037 1228.5058
1397.8829 1398.9714 1417.6648
1466.1660 1564.4023 1704.4184
3210.2306 3213.9147 3227.8954
3230.7260 3240.2028 3243.0939

wB97XD/aug-cc-pVTZ, B3G optimum
Electronic energy (in Hartree):
-231.908595036
Structure (in Angstrom):
C 0.000000 -0.000000 1.430447
C 0.000000 1.184412 0.722341
C 0.000000 1.184412 -0.722341
C 0.000000 -0.000000 -1.430447
C -0.000000 -1.184412 -0.722341
C -0.000000 -1.184412 0.722341
H 0.000000 2.135106 -1.238283
H -0.000000 0.000000 -2.509927
H -0.000000 -2.135106 -1.238283
H -0.000000 -2.135106 1.238283
H -0.000000 0.000000 2.509927
H -0.000000 2.135106 1.238283
Frequencies (in cm-1):
i592.1426 292.7704 322.3773
383.4990 605.8717 692.5648
854.4509 854.5042 892.7034
962.7328 990.0589 1018.0519
1018.6813 1021.0411 1025.1590
1095.0116 1208.8603 1215.7732
1349.1402 1398.2732 1413.3880
1477.4493 1546.9635 1606.7471
3214.0349 3219.1295 3223.8917
3227.3387 3241.6043 3243.9536

wB97XD/aug-cc-pVTZ, D2 optimum, C2v
Electronic energy (in Hartree):
-231.889764195
Structure (in Angstrom):
6 1.219261 0.715408 -0.004563
6 0.000000 1.317811 0.007747
6 -1.219261 0.715408 -0.004563
6 -1.219261 -0.715408 -0.004563
6 -0.000000 -1.317811 0.007747

6 1.219261 -0.715408 -0.004563
1 -2.184945 1.204450 -0.047548
1 -2.184945 -1.204450 -0.047548
1 0.000000 -2.428562 0.103364
1 2.184945 -1.204450 -0.047548
1 2.184945 1.204450 -0.047548
1 0.000000 2.428562 0.103364
Frequencies (in cm-1):
107.7330 161.0286 394.4024
533.5866 579.5287 668.3243
696.7026 720.8748 811.2625
880.9125 884.3681 955.5399
959.2669 1011.4909 1014.4706
1047.1143 1109.4044 1166.5893
1247.0632 1257.6806 1388.4482
1403.5459 1512.6988 2708.8091
2876.2540 3123.6269 3166.9580
3200.3240 3209.8178 3915.8196

wB97XD/aug-cc-pVTZ, D2 optimum, D2h
Electronic energy (in Hartree):
-231.890058676
Structure (in Angstrom):
6 0.000000 1.219022 0.715453
6 0.000000 -0.000000 1.315336
6 -0.000000 -1.219022 0.715453
6 -0.000000 -1.219022 -0.715453
6 0.000000 -0.000000 -1.315336
6 0.000000 1.219022 -0.715453
1 0.000000 -2.186535 1.202852
1 0.000000 -2.186535 -1.202852
1 0.000000 0.000000 -2.431262
1 0.000000 2.186535 -1.202852
1 0.000000 2.186535 1.202852
1 -0.000000 0.000000 2.431262
Frequencies (in cm-1):
i153.0048 108.2795 387.9335
531.2854 558.6834 672.2969
682.5832 688.2570 777.2655
871.1485 900.1259 924.1382
958.0615 1014.7119 1017.9199
1047.6072 1096.6149 1166.3292
1242.0846 1270.1411 1388.1602
1392.7760 1522.7515 2690.1395
2873.8179 3115.8854 3165.3764

3199.5744 3210.1742 3526.5363

BHandHLYP/cc-pVDZ, B2G optimum
Electronic energy (in Hartree):
-231.791704390
Structure (in Angstrom):
C -0.000000 -0.000000 1.375179
C -0.000000 1.243534 0.680946
C 0.000000 1.243534 -0.680946
C 0.000000 -0.000000 -1.375179
C -0.000000 -1.243534 -0.680946
C -0.000000 -1.243534 0.680946
H 0.000000 2.167928 -1.245480
H 0.000000 0.000000 -2.460632
H -0.000000 -2.167928 -1.245480
H -0.000000 -2.167928 1.245480
H -0.000000 0.000000 2.460632
H -0.000000 2.167928 1.245480
Frequencies (in cm-1):
260.4783 308.0925 361.0422
482.5146 617.2279 708.5366
840.2688 942.6854 995.5966
1006.9614 1016.5066 1038.6712
1046.1329 1068.0717 1077.3660
1104.8062 1225.0452 1245.6481
1415.9479 1421.1023 1463.0818
1492.7935 1600.8104 1756.4548
3295.3566 3299.5883 3311.5785
3315.1189 3324.5667 3327.4030

BHandHLYP/cc-pVDZ, B3G optimum
Electronic energy (in Hartree):
-231.791506317
Structure (in Angstrom):
C -0.000000 -0.000000 1.432062
C 0.000000 1.186648 0.722360
C 0.000000 1.186648 -0.722360
C 0.000000 -0.000000 -1.432062
C -0.000000 -1.186648 -0.722360
C -0.000000 -1.186648 0.722360
H 0.000000 2.139734 -1.240061
H 0.000000 0.000000 -2.514336
H -0.000000 -2.139734 -1.240061
H -0.000000 -2.139734 1.240061
H -0.000000 0.000000 2.514336

H -0.000000 2.139734 1.240061
Frequencies (in cm-1):
i303.8980 323.6011 347.3768
460.8501 618.1920 708.3004
883.1212 923.1148 923.3583
987.2050 1016.0762 1034.1576
1054.6559 1056.4388 1064.7421
1116.1178 1224.1718 1235.0343
1385.8828 1420.8629 1459.8312
1507.1448 1580.5700 1657.1799
3298.2057 3303.7596 3308.3986
3312.0753 3326.5473 3328.8184

BHandHLYP/cc-pVDZ, D2 optimum, C2v
Electronic energy (in Hartree):
-231.773285117
Structure (in Angstrom):
6 1.225939 0.717344 -0.019620
6 0.000000 1.363719 0.039638
6 -1.225939 0.717344 -0.019620
6 -1.225939 -0.717344 -0.019620
6 -0.000000 -1.363719 0.039638
6 1.225939 -0.717344 -0.019620
1 -2.169992 1.233287 -0.161936
1 -2.169992 -1.233287 -0.161936
1 0.000000 -2.427706 0.321484
1 2.169992 -1.233287 -0.161936
1 2.169992 1.233287 -0.161936
1 0.000000 2.427706 0.321484
Frequencies (in cm-1):
111.2373 430.5911 458.6327
600.0095 622.4106 681.0901
758.3284 803.3727 849.1280
871.2649 942.4110 964.3566
1038.6793 1079.4975 1118.3438
1121.3398 1163.9823 1178.4857
1245.8971 1327.9087 1417.3232
1466.1851 1507.9741 3059.3634
3060.1329 3217.8048 3272.0360
3293.2654 3294.2423 3310.8974

BHandHLYP/cc-pVDZ, D2 optimum, D2h
Electronic energy (in Hartree):
-231.772371637
Structure (in Angstrom):

6 -0.000000 1.221195 0.717557
6 -0.000000 0.000000 1.319535
6 -0.000000 -1.221195 0.717557
6 -0.000000 -1.221195 -0.717557
6 0.000000 0.000000 -1.319535
6 0.000000 1.221195 -0.717557
1 -0.000000 -2.193157 1.202744
1 0.000000 -2.193157 -1.202744
1 0.000000 -0.000000 -2.438036
1 0.000000 2.193157 -1.202744
1 0.000000 2.193157 1.202744
1 -0.000000 -0.000000 2.438036
Frequencies (in cm-1):
i696.3548 137.9491 417.9135
532.5596 577.1048 683.3464
704.8301 706.1318 733.4684
844.2982 919.6711 968.2345
972.7034 1010.0015 1035.7734
1072.5678 1111.8732 1186.6994
1250.1665 1258.3875 1344.8976
1403.1479 1423.4807 1564.8001
2753.2865 2937.9418 3197.5584
3239.9329 3274.9948 3287.6503

BHandHLYP/aug-cc-pVTZ, B2G optimum
Electronic energy (in Hartree):
-231.855641029
Structure (in Angstrom):
C -0.000000 0.000000 1.367593
C -0.000000 1.237481 0.676688
C 0.000000 1.237481 -0.676688
C 0.000000 -0.000000 -1.367593
C -0.000000 -1.237481 -0.676688
C -0.000000 -1.237481 0.676688
H 0.000000 2.152855 -1.236240
H 0.000000 0.000000 -2.442469
H -0.000000 -2.152855 -1.236240
H -0.000000 -2.152855 1.236240
H -0.000000 0.000000 2.442469
H -0.000000 2.152855 1.236240
Frequencies (in cm-1):
265.0259 295.7659 355.6465
467.3785 621.4358 714.6767
845.4779 941.8782 994.0989
1008.6931 1012.1739 1047.1219

1051.2797 1072.4406 1073.8547
1105.5854 1238.5458 1258.7751
1429.8334 1444.5748 1445.9259
1511.0213 1607.2475 1747.7221
3281.9431 3286.1518 3297.1146
3300.7573 3309.4447 3312.3374

BHandHLYP/aug-cc-pVTZ, B3G optimum
Electronic energy (in Hartree):
-231.855423139
Structure (in Angstrom):
C 0.000000 0.000000 1.424850
C -0.000000 1.179767 0.719234
C 0.000000 1.179767 -0.719234
C 0.000000 0.000000 -1.424850
C -0.000000 -1.179767 -0.719234
C -0.000000 -1.179767 0.719234
H -0.000000 2.123890 -1.231495
H 0.000000 0.000000 -2.496936
H -0.000000 -2.123890 -1.231495
H -0.000000 -2.123890 1.231495
H -0.000000 0.000000 2.496936
H -0.000000 2.123890 1.231495
Frequencies (in cm-1):
i316.9508 313.0153 341.1099
445.0141 622.9471 714.5850
884.5854 922.4848 929.6115
984.3981 1011.6587 1047.3901
1057.8824 1057.9133 1058.7186
1119.2541 1237.2377 1244.5903
1401.7494 1444.9300 1445.8814
1515.8545 1591.6577 1650.3316
3284.3739 3289.7112 3294.3719
3298.0139 3311.3031 3313.6263

BHandHLYP/aug-cc-pVTZ, D2 optimum, C2v
Electronic energy (in Hartree):
-231.836659647
Structure (in Angstrom):
6 1.218569 0.714316 -0.019317
6 0.000000 1.353700 0.038736
6 -1.218569 0.714316 -0.019317
6 -1.218569 -0.714316 -0.019317
6 -0.000000 -1.353700 0.038736
6 1.218569 -0.714316 -0.019317

1 -2.155262 1.222671 -0.159466
1 -2.155262 -1.222671 -0.159466
1 0.000000 -2.409074 0.318325
1 2.155262 -1.222671 -0.159466
1 2.155262 1.222671 -0.159466
1 0.000000 2.409074 0.318325
Frequencies (in cm-1):
136.9605 430.1031 459.6472
611.5968 623.4298 689.3568
775.2310 816.3700 867.5672
875.1205 948.3467 977.8270
1050.4243 1087.6562 1127.9737
1142.1269 1164.8457 1188.6581
1198.6321 1346.8100 1398.7399
1451.5763 1513.4273 3025.8015
3031.2107 3080.5850 3229.8683
3256.8617 3279.8060 3279.9425

BHandHLYP/aug-cc-pVTZ, D2 optimum, D2h
Electronic energy (in Hartree):
-231.835984685
Structure (in Angstrom):
6 0.000000 1.213856 0.714907
6 -0.000000 0.000000 1.312556
6 -0.000000 -1.213856 0.714907
6 -0.000000 -1.213856 -0.714907
6 0.000000 0.000000 -1.312556
6 0.000000 1.213856 -0.714907
1 0.000000 -2.177081 1.194738
1 0.000000 -2.177081 -1.194738
1 0.000000 -0.000000 -2.421567
1 0.000000 2.177081 -1.194738
1 0.000000 2.177081 1.194738
1 -0.000000 -0.000000 2.421567
Frequencies (in cm-1):
i656.8804 170.1653 412.6837
538.2807 582.3291 677.0182
689.9397 712.7112 717.7989
860.9824 912.2466 970.1222
976.6022 1019.9046 1028.5963
1077.5940 1119.8256 1193.9762
1274.0505 1281.0145 1382.7067
1383.3878 1427.5477 1553.8450
2738.7439 2922.9114 3185.1097
3226.4632 3262.1996 3274.6125

MRCI(3,4)/def2TZVP, B2G optimum
Electronic energy (in Hartree):
-231.25442449
Structure (in Angstrom):
C 0.0004329607 1.3695577628 0.0000000000
C 1.2391497639 0.6764167807 0.0000000000
C 1.2391497636 -0.6764167749 0.0000000000
C 0.0004329499 -1.3695577621 0.0000000000
C -1.2399862107 -0.6809798036 0.0000000000
C -1.2399862037 0.6809797955 0.0000000000
H 2.1555262776 -1.2376323038 0.0000000000
H 0.0014000248 -2.4462488535 0.0000000000
H -2.1565228116 -1.2419919483 0.0000000000
H -2.1565227728 1.2419919693 0.0000000000
H 0.0013999573 2.4462488528 0.0000000000
H 2.1555263009 1.2376322850 0.0000000000
Frequencies (in cm-1):
309.93 359.78 503.00 622.47 642.82
721.08 856.48 957.00 1015.34 1023.17
1048.15 1057.84 1058.54 1080.74 1118.24
1249.29 1268.90 1292.84 1462.63 1494.64
1520.11 1625.91 1774.26 1852.24 3314.43
3316.12 3325.93 3329.75 3338.10 3341.61

MRCI(3,4)/def2TZVP, B3G optimum
Electronic energy (in Hartree):
-231.25435411
Structure (in Angstrom):
C 0.0000000219 1.4259723431 0.0000000000
C 1.1793117774 0.7199359291 0.0000000000
C 1.1846398533 -0.7199555121 0.0000000000
C -0.0000000223 -1.4294892818 0.0000000000
C -1.1846398678 -0.7199555315 0.0000000000
C -1.1793117797 0.7199359040 0.0000000000
H 2.1312649647 -1.2309699650 0.0000000000
H -0.0000000109 -2.5033491444 0.0000000000
H -2.1312649679 -1.2309699905 0.0000000000
H -2.1246363231 1.2345577971 0.0000000000
H 0.0000000448 2.4997296116 0.0000000000
H 2.1246363096 1.2345578402 0.0000000000
Frequencies (in cm-1):
264.13 325.82 346.58 482.22 618.32
721.08 892.31 942.08 996.61 1015.72
1018.12 1033.61 1044.14 1069.10 1073.94

1126.54 1247.10 1255.76 1424.32 1449.03
1488.22 1524.33 1602.17 1672.69 3310.71
3316.07 3321.86 3326.07 3340.10 3342.86

CCSD(T)/aug-cc-pVTZ//ωB97XD/aug-cc-pVTZ, C2H2+
Electronic energy (in Hartree):
-0.76775848331D+02
Structure (in Angstrom):
C 0.000000 0.000000 0.620148
C 0.000000 0.000000 -0.620148
H 0.000000 0.000000 1.699002
H 0.000000 0.000000 -1.699002

CCSD(T)/aug-cc-pVTZ//ωB97XD/aug-cc-pVTZ, C2H2
Electronic energy (in Hartree):
-0.77191697635D+02
Structure (in Angstrom):
C 0.000000 0.000000 0.597039
C 0.000000 0.000000 -0.597039
H 0.000000 0.000000 1.659109
H 0.000000 0.000000 -1.659109

CCSD(T)/aug-cc-pVTZ//ωB97XD/aug-cc-pVTZ, C4H4+
Electronic energy (in Hartree):
-0.15402973796D+03
Structure (in Angstrom):
C -1.553569 0.399287 -0.125356
C -0.729695 -0.398898 0.373474
H -0.781583 -1.134274 1.165880
H -2.141554 1.131946 -0.643416
C 0.729697 -0.398897 -0.373481
H 1.553571 0.399261 0.125390
H 2.141520 1.132080 0.643265
H 0.781593 -1.134267 -1.165891

CCSD(T)/aug-cc-pVTZ//ωB97XD/aug-cc-pVTZ, Min 1
Electronic energy (in Hartree):
-0.23131675685D+03
Structure (in Angstrom):
C -1.496756 -1.091458 -0.000087
C -1.577839 0.233761 0.000332
H -2.560577 0.703034 0.001001
H -2.190968 -1.920156 -0.000013
C -0.452713 1.107555 -0.000176
C 0.905147 0.790599 -0.000287

H 1.609534 1.610701 -0.000657
H -0.687028 2.168497 -0.000509
C 1.377793 -0.579493 0.626098
C 1.377765 -0.579825 -0.625896
H 1.512953 -0.925259 -1.634028
H 1.515712 -0.923648 1.634299

CCSD(T)/aug-cc-pVTZ//ωB97XD/aug-cc-pVTZ, TS 1
Electronic energy (in Hartree):
-0.23131304286D+03
Structure (in Angstrom):
C -1.173651 -1.126102 -0.040425
C -1.537306 0.139567 0.159052
H -2.541401 0.416842 0.466705
H -1.621170 -2.101853 0.089275
C -0.559731 1.134829 -0.058651
C 0.798140 0.804716 -0.217769
H 1.469919 1.576056 -0.571976
H -0.857939 2.176344 -0.120808
C 1.469083 -0.315211 0.646010
C 1.073626 -0.703834 -0.500507
H 1.124288 -1.340454 -1.366441
H 2.005330 -0.330725 1.576983

CCSD(T)/aug-cc-pVTZ//ωB97XD/aug-cc-pVTZ, Min 2
Electronic energy (in Hartree):
-0.23139096613D+03
Structure (in Angstrom):
C 0.691553 -0.748576 -0.403476
C 0.691362 0.749013 -0.403063
C 1.323043 -0.000178 0.742085
C -0.666264 -1.117425 -0.030316
C -1.465415 -0.000209 0.137072
C -0.666612 1.117327 -0.030218
H -2.489854 -0.000483 0.472214
H -0.966787 -2.135886 0.180237
H 1.324185 -1.371797 -1.020143
H 2.329970 -0.000173 1.134190
H 1.324023 1.372895 -1.019016
H -0.967547 2.135733 0.180010

CCSD(T)/aug-cc-pVTZ//ωB97XD/aug-cc-pVTZ, TS 2
Electronic energy (in Hartree):
-0.23138474979D+03
Structure (in Angstrom):

```
C -0.714883 -0.743245 0.418454
C -0.650324 0.908171 0.148407
C -1.474974 -0.118983 -0.571078
C 0.667709 -1.132971 -0.000783
C 1.492732 -0.074600 -0.137707
C 0.740124 1.109108 0.036477
H 2.530049 -0.105678 -0.430517
H 0.884485 -2.163259 -0.248089
H -1.139082 -1.138963 1.338519
H -2.539835 -0.051443 -0.739628
H -1.263029 1.672252 0.612067
H 1.165111 2.102217 0.105026
```

CCSD(T)/aug-cc-pVTZ//ωB97XD/aug-cc-pVTZ, Min 3
Electronic energy (in Hartree):
-0.23147949977D+03
Structure (in Angstrom):

```
C 0.000207 -1.372988 0.000098
C -1.242292 0.679677 -0.000173
C -1.242073 -0.680010 0.000035
C 1.242287 -0.679684 -0.000082
C 1.242078 0.680008 -0.000070
C -0.000208 1.372994 0.000118
H 2.163815 1.243382 0.000008
H 2.164214 -1.242735 -0.000234
H 0.000310 -2.455464 0.000232
H -2.163816 -1.243379 0.000199
H -2.164204 1.242746 -0.000476
H -0.000309 2.455465 0.000712
```

References

(1) Tachikawa, H. Jahn–teller effect of the benzene radical cation: A direct ab initio molecular dynamics study. *J. Phys. Chem. A* **2018**, *122*, 4121–4129.

(2) Vidal, M. L.; Epshtein, M.; Scutelnic, V.; Yang, Z.; Xue, T.; Leone, S. R.; Krylov, A. I.;

Coriani, S. Interplay of open-shell spin-coupling and Jahn–Teller distortion in benzene radical cation probed by X-ray spectroscopy. *J. Phys. Chem. A* **2020**, *124*, 9532–9541.

(3) Werner, H.-J.; Knowles, P. J.; Knizia, G.; Manby, F. R.; Schütz, M. Molpro: a general-purpose quantum chemistry program package. *Wiley Interdiscip. Rev. Comput. Mol. Sci.* **2012**, *2*, 242–253.
